# Conserved and non-conserved functions of the rice homologs of the Arabidopsis trichome initiation-regulating MBW complex proteins

**DOI:** 10.1186/s12870-021-03035-0

**Published:** 2021-05-25

**Authors:** Kaijie Zheng, Xutong Wang, Yating Wang, Shucai Wang

**Affiliations:** 1 Key Laboratory of Soybean Molecular Design Breeding, Northeast Institute of Geography and Agroecology, Chinese Academy of Sciences, Changchun, China; 2 Key Laboratory of Molecular Epigenetics of MOE, Institute of Genetics and Cytology, Northeast Normal University, Changchun, China; 3 Laboratory of Plant Molecular Genetics & Crop Gene Editing, School of Life Sciences, Linyi University, Linyi, China

**Keywords:** MBW complex, Trichome initiation, Transcription factor, Rice, Arabidopsis

## Abstract

**Background:**

Trichome initiation in Arabidopsis is regulated by a MYB-bHLH-WD40 (MBW) transcriptional activator complex formed by the R2R3 MYB transcription factor GLABRA1 (GL1), MYB23 or MYB82, the bHLH transcription factor GLABRA3 (GL3), ENHANCER OF GLABRA3 (EGL3) or TRANSPARENT TESTA8 (TT8), and the WD40-repeat protein TRANSPARENT TESTA GLABRA1 (TTG1). However, the functions of the rice homologs of the MBW complex proteins remained uncharacterized.

**Results:**

Based on amino acid sequence identity and similarity, and protein interaction prediction, we identified OsGL1s, OsGL3s and OsTTG1s as rice homologs of the MBW complex proteins. By using protoplast transfection, we show that OsGL1D, OsGL1E, OsGL3B and OsTTG1A were predominantly localized in the nucleus, OsGL3B functions as a transcriptional activator and is able to interact with GL1 and TTG1. By using yeast two-hybrid and protoplast transfection assays, we show that OsGL3B is able to interact with OsGL1E and OsTTG1A, and OsGL1E and OsTTG1A are also able to interact with GL3. On the other hand, we found that OsGL1D functions as a transcription activator, and it can interact with GL3 but not OsGL3B. Furthermore, our results show that expression of *OsTTG1A* in the *ttg1* mutant restored the phenotypes including alternations in trichome and root hair formation, seed color, mucilage production and anthocyanin biosynthesis, indicating that OsTTG1A and TTG1 may have similar functions.

**Conclusion:**

These results suggest that the rice homologs of the Arabidopsis MBW complex proteins are able to form MBW complexes, but may have conserved and non-conserved functions.

**Supplementary Information:**

The online version contains supplementary material available at 10.1186/s12870-021-03035-0.

## Background

Trichomes are appendages on the surfaces of the aerial parts of the plants. Trichomes are developed from epidermal cells and are diverse in appearance. Trichomes can protect plants from excessive heat and water loss, and from insect or pathogen attacks by increasing the boundary layer thickness between the epidermal tissues and the environment [1, 2].

Available evidence suggests that trichome initiation in Arabidopsis is regulated by a MYB-bHLH-WD40 (MBW) complex formed by a R2R3 MYB transcriptional activator, a bHLH transcription factor, and a WD40-repeat protein [3–7]. The R2R3 MYB transcription factor in this MBW complex is GLABRA1 (GL1) [8], the bHLH transcription factor is GLABRA3 (GL3), ENHANCER OF GLABRA3 (EGL3) [9, 10], or TRANSPARENT TESTA8 (TT8) [11], and the WD40-repeat protein is TRANSPARENT TESTA GLABRA1 (TTG1) [12]. It has been shown that MYB23 and MYB82 are also able to interact with GL3 and/or EGL3, and to regulate trichome formation [13, 14]. The MBW transcriptional activator complex is able to induce the expression of the homeodomain protein gene *GLABRA2* (*GL2*) [15], leading to the promotion of trichome initiation [3–7, 16].

This MBW complex is also able to induce the expression of some R3 MYB genes including *TRYPTICHON* (*TRY*), *CAPRICE* (*CPC*), *ENHANCER OF TRY AND CPC1* (*ETC1*) and *ETC3* [17–22]. These R3 MYB transcriptional factors, including ETC2, TRICHOMELESS1 (TCL1) and TCL2, whose expression are not regulated by the MBW complex [22–25], are able to move to their neighboring cells, where they competing with GL1 for binding of GL3, therefore inhibiting the formation of the MBW complex, leading to the inhibition of trichome initiation [3–7, 26–28].

At least in some plants, functions of MBW complex proteins in regulating trichome initiation is conserved, for example, *Brassica napus* plants expressing Arabidopsis *GL3* produced ectopic trichomes [29], cotton homologs of GL1 and GL2 regulate trichome initiation in Arabidopsis [30, 31], and trichome phenotypes in the *ttg1* mutants were restored by expressing an apple *TTG1* homolog gene [32].

Even though trichomes could provide protection for plants [1, 2], glabrous has been considered to be a favorite agronomic trait in rice (*Oryza sativa*), because grains of glabrous rice have greater packing capability, and glabrous rice produces less itchy causing dust [33, 34]. Therefore, great efforts have been devoted to addressing the regulation mechanisms under the control of trichome initiation in rice.

So far, several regulator of trichome initiation have been identified in rice, some of them are homologs of Arabidopsis trichome initiation regulators, whereas others are not. For example, OsWOX3B, a homolog of GL2, regulates trichome initiation in rice [33–35]. SPL9 is a squamosa promoter binding type protein that has been shown to regulate trichome initiation in Arabidopsis via directly regulating the expression of *TCL1* [36], OsSPL10, a homolog of SPL9 is also able to regulate trichome initiation in rice [37]. These results suggest that trichome initiation in rice may be regulated by similar mechanisms as in Arabidopsis.

On the other hand, SDG714, a histone H3K9 Methyltransferase, HL6 (Hairy Leaf 6), an AP2/ERF transcription factor and type-B response regulators have been shown to be involved in the regulation of trichome initiation in rice [35, 38, 39], but none of them are homologs of known Arabidopsis trichome initiation regulators. Our previous studies have also shown that OsTCL1, a homolog of TCL1, is able to regulate trichome initiation in Arabidopsis, but not in rice [40]. These results indicate that trichome initiation in rice may also be regulated by different mechanisms.

Here we report the identification and characterization of rice homologs of the Arabidopsis trichome initiation-regulating MBW complex proteins. Based on amino acid sequence identity and similarity, and protein interaction prediction, we identified OsGL1A—OsGL1E, OsGL3A—OsGL3C, and OsTTG1A and OsTTG1B as homologs of GL1, GL3 and TTG1, respectively. By using Arabidopsis protoplast transfection assays, we found that these proteins may have conserved and non-conserved functions in forming MBW complexes. By generating transgenic plants expressing *OsTTG1A* in the *ttg1* mutants, we show that OsTTG1A and TTG1 may have similar functions in regulating trichome initiation as well as root hair formation and secondary metabolism in Arabidopsis.

## Results

### Homologs of the MBW complex proteins in rice

In previous experiments, we have identified *OsGL1A*, *OsGL1B* and *OsGL1C* as rice homologs of Arabidopsis GL1, *OsGL3A*, *OsGL3B* and as rice homologs of Arabidopsis GL3, and *OsTTG1A*, and *OsTTG1B* as rice homologs of Arabidopsis TTG1[40].

**Fig. 1:**
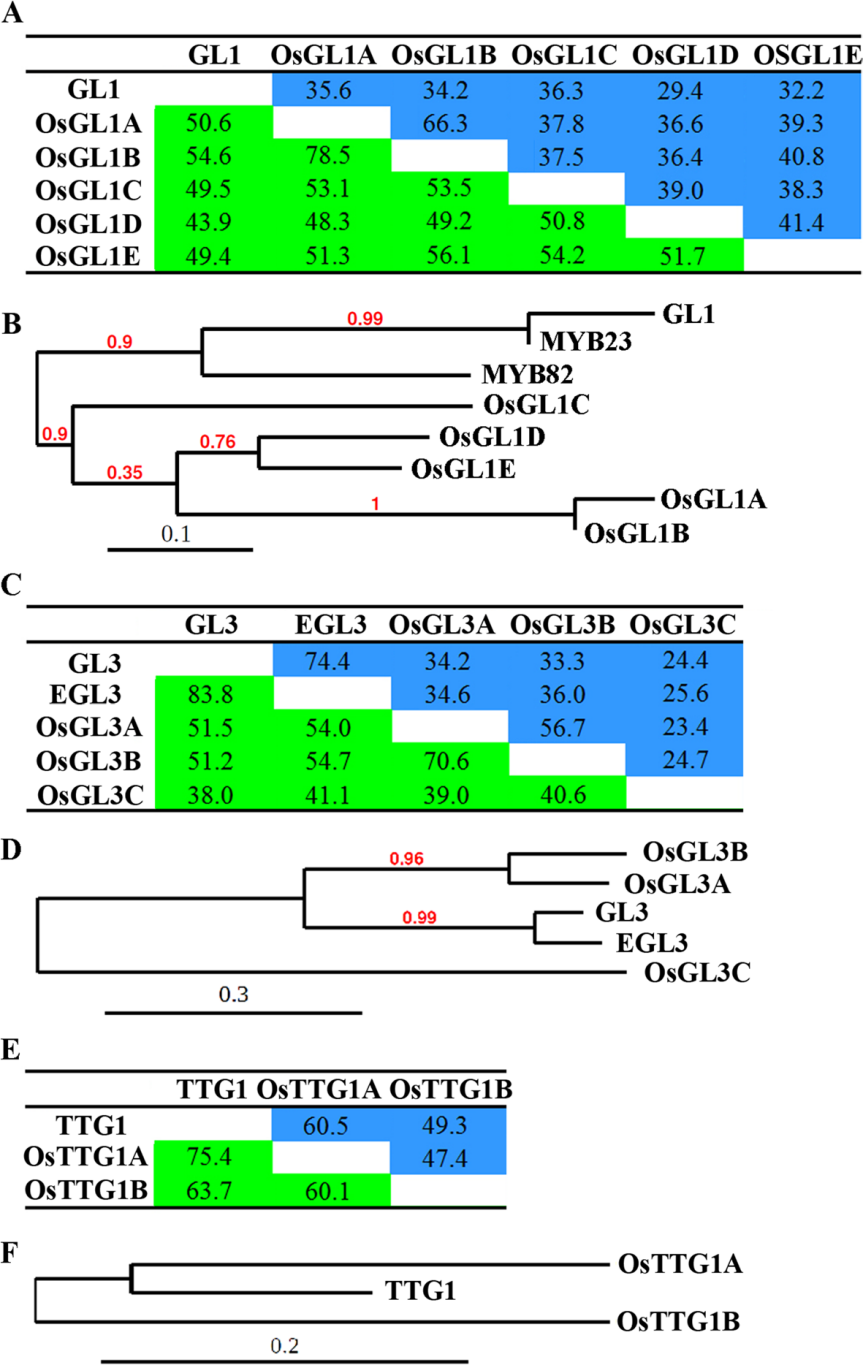
GL1, GL3 and TTG1 homologs in rice. **a** Amino acid identity and similarity of GL1 and OsGL1s. OsGL1s, homologs of GL1 were identified by using “Protein Homologs” on Phytozome (https://phytozome.jgi.doe.gov/pz/portal.html#). Percentages of amino acid similarity and identity of GL1 and OsGL1s were calculated by using MatGAT (v2.02). Percentage of amino acid identity are shaded in green, and percentage of amino acid similarity in blue. **b** Phylogenetic tree of GL1 and OsGL1s. The entire amino acid sequences of GL1 and OsGL1s were used for phylogenetic analysis on Phylogeny (www.phylogeny.fr) by using “One Click” mode with default settings. The number above the branch indicates branch support values. Bar indicates branch length. **c** Amino acid identity and similarity of GL3 and OsGL3s. OsGL3s were identified by using “Protein Homologs” on Phytozome (https://phytozome.jgi.doe.gov/pz/portal.html#). Percentages of amino acid similarity and identity of GL3 and OsGL3s were calculated by using MatGAT (v2.02). Percentage of amino acid identity are shaded in green, and percentage of amino acid similarity in blue. **d** Phylogenetic tree of GL3 and OsGL3s. The entire amino acid sequences of GL3 and OsGL3s were used for phylogenetic analysis on Phylogeny (www.phylogeny.fr) by using “One Click” mode with default settings. The number above the branch indicates branch support values. Bar indicates branch length. **e** Amino acid identity and similarity of TTG1 and OsTTG1s. OsTTG1s were identified by using “Protein Homologs” on Phytozome (https://phytozome.jgi.doe.gov/pz/portal.html#). Percentages of amino acid similarity and identity of TTG1 and OsTTG1s were calculated by using MatGAT (v2.02). Percentages of amino acid identity are shaded in green, and percentage of amino acid similarity in blue. **f** Phylogenetic tree of TTG1 and OsTTG1s. The entire amino acid sequences of TTG1 and OsTTG1s were used for phylogenetic analysis on Phylogeny (www.phylogeny.fr) by using “One Click” mode with default settings. The number above the branch indicates branch support values. Bar indicates branch length

To examine if these MBW homologs in rice can form MBW complexes, we first analyzed their interaction relationship on STRING (https://string-db.org/). We found that OsGL1D (Loc_Os03g29614) and OsGL1E (Loc_Os06g10350) were predicted as potential interaction proteins of OsGL3B. As shown in Fig. 1a, OsGL1 proteins showed a 29.4% ~ 36.3% identity, and a 43.9% ~ 54.6% similarity with GL1 at amino acid level (Fig. 1a). Phylogenetic analysis showed that OsGL1A is closely related to OsGL1B, whereas OsGL1D is closed related to OsGL1E. Together with OsGL1C, these five OsGL1s formed a clade (Fig. 1b). On the other hand, GL1 is closed related to MYB23, and they formed another clade together with MYB82 (Fig. 1b). Sequence alignment showed that the most conserved region of the OsGL1s is the R2R3 MYB domain (Fig. S1). The [D/E]L × 2[R/K] × 3L × 6L × 3R amino acid signature required for the interaction of MYB transcription factors with R/B-like bHLH transcription factors [41], and the S residue has been shown to be required for the activation of *GL2* [42], are fully conserved in all the five OsGL1s (Fig. S1).

As for the OsGL3s, both OsGL3A and OsGL3B showed a more than 33% identity and a more than 51% similarity with GL3 and EGL3, whereas that for OsGL3C are only about 25% and 40%, respectively (Fig. 1c). Phylogenetic analysis showed that OsGL3A is closely related to OsGL3B, and they formed a clade with GL3 and EGL3 pair (Fig. 1d). Sequence alignment showed that the most conserved regions of the OsGL3s are the N-terminal and C-terminal domains (Fig. S2). OsGL3A and OsGL3B, but not OsGL3C showed high similarity to GL3 and EGL3 at the HLH domain region and the first 97 amino acids required for GL3 to interact with GL1 [9] (Fig. S2).

Among the MBW complex protein homologs in rice, OsTTG1s are the most conserved ones when compared with their Arabidopsis homologs. OsTTG1A and OsTTG1B showed a 60.5% and 49.3% identity, and a 75.4% and 63.7% similarity, respectively to TTG1 (Fig. 1e). Phylogenetic analysis showed that OsTTG1A is closely related to TTG1 (Fig. 1f). Sequence alignment showed OsTTG1s and TTG1 are highly conserved at full-length amino acid sequence level (Fig. S3), including the 25 amino acid sequence that is required for interaction of TTG1 with GL3 [9].

In order to get a better pictures on the relations that exists between the Arabidopsis MBW complex component proteins and their rice homologs, we identified MBW complex component protein homologs, i.e., proteins with highest amino acid similarity with GL1, GL3 and TTG1, respectively, in the Brassicaceae family plants *Brassica rapa*, *Capsella grandiflora* and *Capsella rubella*, the Fabidae family plant *Glycine max*, the Malpighiales family plant *Populus trichocarpa*, and the Panicoideae family plants *Zea mays*, *Setaria italica* and *Panicum hallii*, and expended the phylogenetic analysis. The results show that OsGL1s and the Arabidopsis GL1, MYB23 and MYB82 are still in two different clades (Fig. S4). The Arabidopsis GL1, MYB23 and MYB82 are closely related to homologs from the three Brassicaceae plants and the Malpighiales family plant *P. trichocarpa*, whereas OsGL1s are closely related to homologs from the three Panicoideae family plants and the Fabidae family plant *G. max* (Fig. S4). On the other hand, OsGL3C and TT8 formed a clade, whereas GL3, EGL3, OsGL3A and OsGL3B formed another clade with homologs from all the eight plants mentioned above, in which OsGL3A and OsGL3B formed a sub-clade with homologs from the three Panicoideae family plants, and GL3 and EGL3 formed another sub-clade with homologs from the three Brassicaceae plants, *P. trichocarpa* and *G. max* (Fig. S5). For the WD40 proteins, OsTTG1B alone formed a clade, whereas OsTTG1A, TTG1 and homologs from all the eight plants formed another clade, in which the OsTTG1A and homologs from the three Panicoideae family plants formed a sub-clade, and TTG1 and homologs from the three Brassicaceae plants, *P. trichocarpa* and *G. max* formed another sub-clade (Fig. S6).

### Subcellular localization of the MBW complex homolog proteins

Previous reports have shown that GL3, GL1 and TTG1 are all localized in the nucleus [43]. Based on the above bioinformatics analysis, OsGL1A, OsGL1B, OsGL1C, OsGL1D, OsGL1E, OsGL3B and OsTTG1A were chosen for subcellular localization assays. OsGL1A and OsGL1B were chosen because they showed relatively high amino acid identity and similarity to GL1. Whereas OsGL1D and OsGL1E are potential interactors of OsGL3B according to STRING assays. OsGL3B was chosen because both OsGL3A and OsGL3B showed relatively high amino acid identity and similarity to GL3, OsGL3B was predicted to interact with OsGL1D and OsGL1E on STRING, whereas OsGL1C is not paired with other OsGL1 proteins. OsTTG1A was chosen because it showed relatively high amino acid identity and similarity to TTG1.

**Fig. 2:**
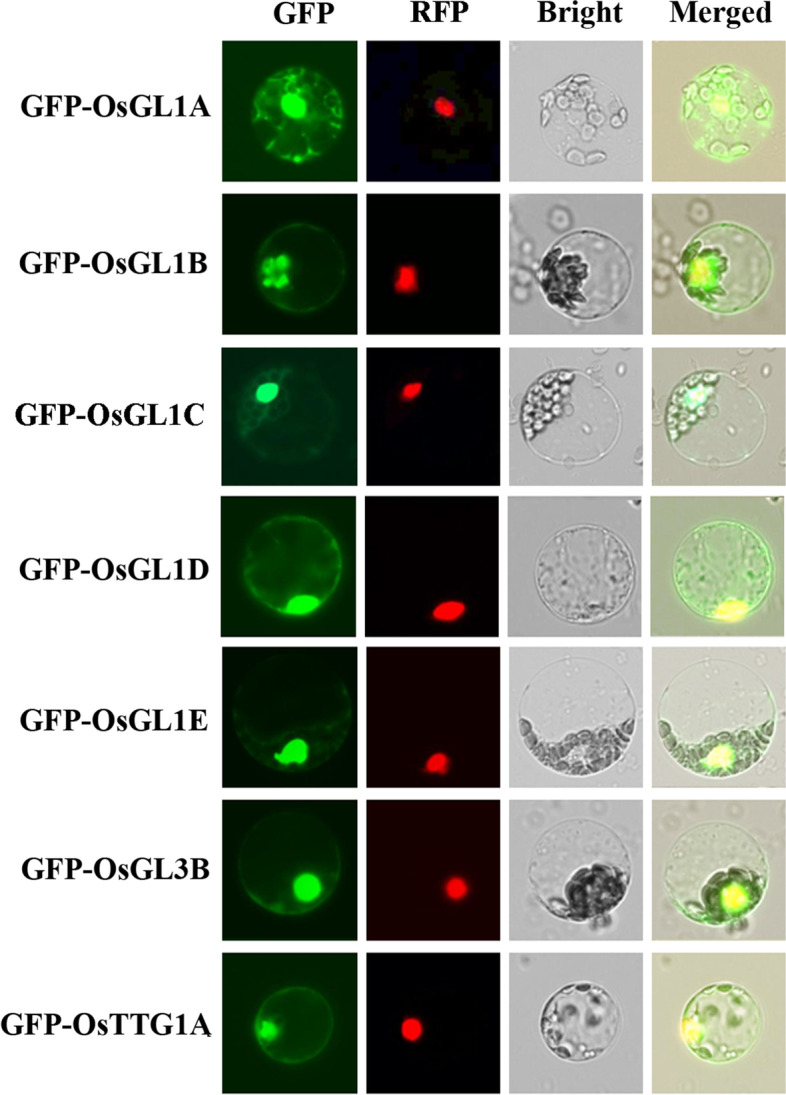
Subcellular localization of OsGL1s, OsGL3B and OsTTG1A in transfected protoplasts. Protoplasts were isolated from rosette leaves of 3 ~ 4-week-old Col wild type plants. Plasmids of *GFP*-*OsGL1A*, *GFP*-*OsGL1B*, *GFP*-*OsGL1D*, *GFP*-*OsGL1E*, *GFP*-*OsGL3B* or *GFP*-*OsTTG1A* were transfected into protoplasts. Plasmids of *35S:NLS-RFP* was co-transfected as a nuclear indicator. After incubated for 20–22 h at room temperature in darkness, GFP and RFP fluorescence were observed and photographed under a fluorescence microscopy

We examined their subcellular localization in Arabidopsis protoplasts. GFP fused constructs of the MBW complex homolog genes were transfected into Arabidopsis protoplasts, and GFP fluorescence was observed under a confocal microscope. We found OsGL1D, OsGL1E, OsGL3B and OsTTG1A were predominantly localized in nucleus, whereas OsGL1A and OsGL1B may be localized in nucleus and likely some other organelles such as cell membranes and chloroplasts (Fig. 2).

### OsGL3B is a transcriptional activator and it interacts with GL1 and TTG1 in Arabidopsis protoplasts

**Fig. 3:**
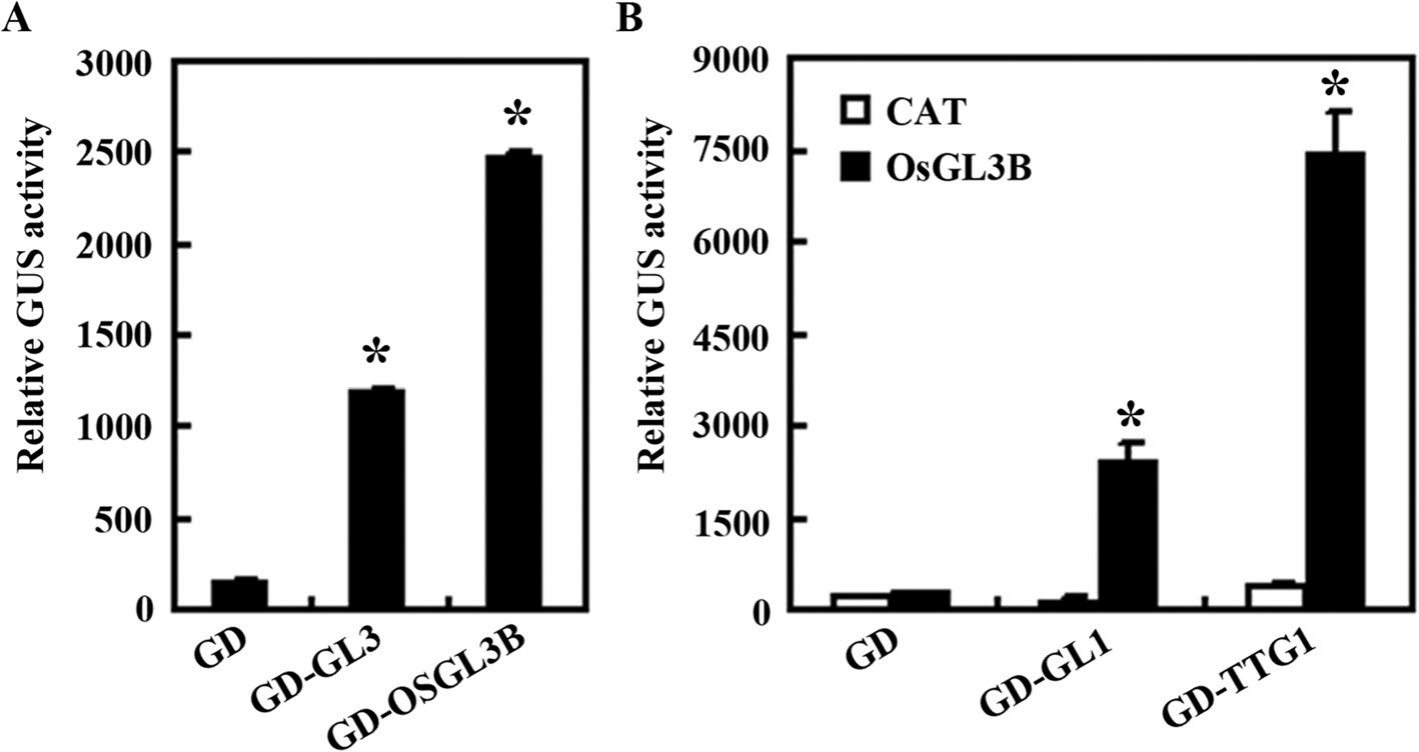
OsGL3B is a transcriptional activator, and it interacts with GL1 and TTG1 in transfected protoplasts. **a** OsGL3B is a transcriptional activator. Plasmids of the *Gal4:GUS* reporter, and *GD*-*OsGL3B* or *GD*-*GL3* effector were co-transfected into protoplasts. Cotransfection of the plasmids of *GD* effector was used as a control. After incubated for 20–22 h at room temperature in darkness, GUS activities were measured by using a microplate reader. Data represent the mean ± SD of three biological replicates. *: Significantly different from the GD control (Student’s *t*-test, *P* < 0.05). **b** OsGL3B is able to interact with GL1 and TTG1. Plasmids of the *Gal4:GUS* reporter, *GD*-*GL1* or *GD*-*TTG1*, and *OsGL3B* or *CAT* effectors were co-transfected into protoplasts. Cotransfection of the plasmids of *GD* effector was used as a control. After incubated for 20–22 h at room temperature in darkness, GUS activities were measured by using a microplate reader. Data represent the mean ± SD of three biological replicates. *: Significantly different from the CAT control (Student’s *t*-test *P* < 0.05)

We have previously shown that GL3 functions as a transcription activator in transfected Arabidopsis protoplasts [44]. To examine if the MBW complex homologs in rice can indeed form MBW complexes, we examined if OsGL3B may also functions as a transcription activator. Plasmids of effector gene *GD*, *GD-OsGL3B* or *GD-GL3*, together with the reporter gene *Gal4-GUS* were co-transfected into Arabidopsis protoplasts, and GUS activities were examined by using a microplate reader. The results show that, similar to *GD-GL3*, cotransfection of *GD-OsGL3B* activated the reporter gene expression (Fig. 3a).

**Fig. 4:**
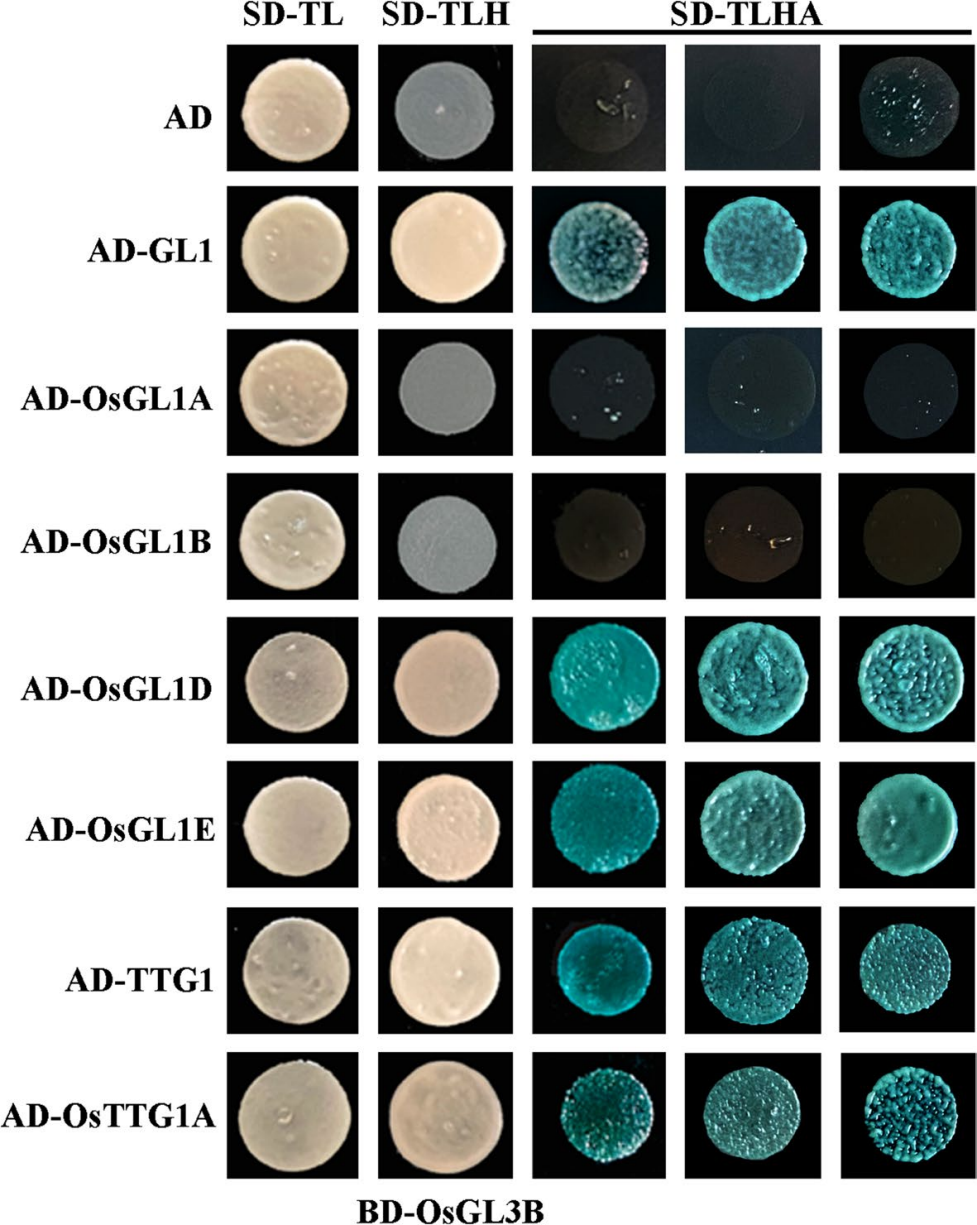
Interaction of OsGL3B with GL1, TTG1 and their rice homologs in yeast cells. Plasmids of the bait and prey constructs were cotransformed into yeast cells and grown in SD-Trp-Leu (SD-TL), SD-Trp-Leu-His (SD-TLH) and SD-Trp-Leu-His-Ade (SD-TLHA). Pictures were taken by using a digital camera

Having shown that OsGL3B functions as a transcriptional activator, we examined if OsGL3B may form a MBW complex with GL1 and TTG1 by examining their interactions in yeast cells and Arabidopsis protoplasts. As shown in Fig. 4, OsGL3B interacted with GL1 and TTG1 in yeast cells. Cotransfection of *OsGL3B* with *GD-GL1* and *GD-TTG1*, respectively activated reporter gene expression in protoplasts (Fig. 3b), indicating that OsGL3B may be able to interact with GL1 and TTG1 in plant cells.

### Interactions of OsTTG1A and OsGL1s with OsGL3B and GL3

**Fig. 5:**
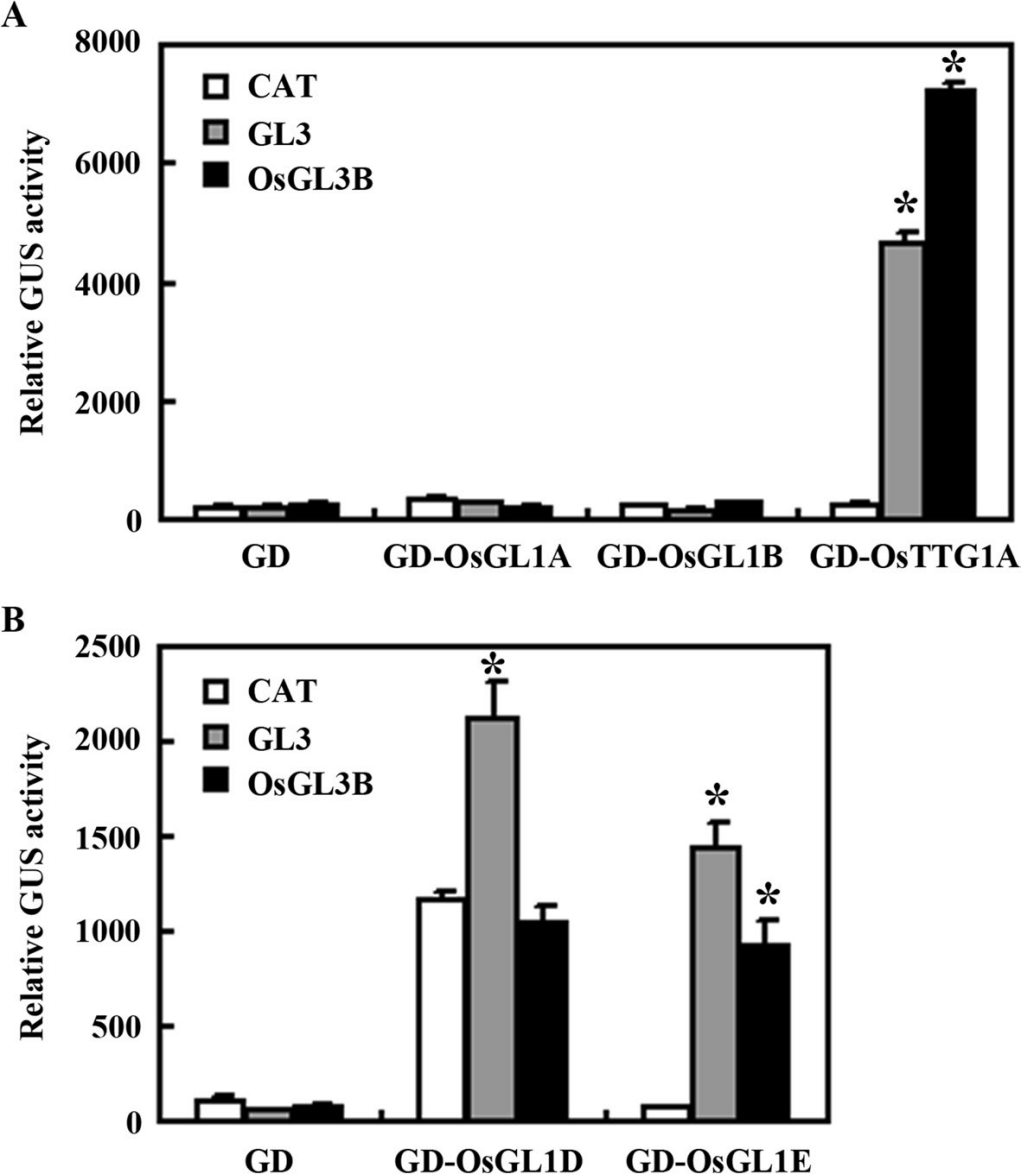
Interaction of OsGL3B with OsGL1s and OsTTG1A in transfected protoplasts. **a** Interaction of OsGL3B with OsGL1A, OsGL1B and OsTTG1A. **b** Interaction of OsGL3B with OsGL1D and OsGL1E. Plasmids of the *Gal4:GUS* reporter, *GD*-*OsGL1s*, and *OsGL3B*, *GL3* or *CAT* effectors were co-transfected into protoplasts. Cotransfection of the plasmids of *GD* effector was used as a control. After incubated for 20–22 h at room temperature in darkness, GUS activities were measured by using a microplate reader. Data represent the mean ± SD of three biological replicates. *: Significantly different from the CAT control according to the Anova with post-hoc Tukey HSD test (https://astatsa.com/OneWay_Anova_with_TukeyHSD/) (*P* < 0.01)

The above results suggest that OsGL3B is able to form a MBW complex with GL1 and TTG1. We then further examined if it may form MBW complex with OsGL1s and OsTTG1A. To do that, we examined interaction of OsGL3B with OsGL1s and OsTTG1A in yeast cells and Arabidopsis protoplasts. As shown in Fig. 4, OsGL3B is able to interact with OsGL1D, OsGL1E and OsTTG1. Similarly, cotransfection of *OsGL3B* with *GD-OsTTG1* activated reporter gene expression in protoplasts, whereas cotransfection of *OsGL3B* with *GD-OsGL1A* or *GD-OsGL1B* failed to do so (Fig. 5a). However, cotransfection of *OsGL3B* with *GD-OsGL1E* activated reporter gene expression (Fig. 5b). These results suggest that OsGL1E, OsGL3B and OsTTG1A can form a MBW complex.

Our protoplast transfection assays also suggest that both OsTTG1A and OsGL1E can interact with GL3 (Fig. 5), indicating that MBW complex proteins in Arabidopsis and rice are interchangeable in forming MBW complex.

Surprisingly, we found that transfection of *GD-OsGL1D* activated reporter gene expression (Fig. 5b), suggesting that unlike GL1 and other OsGL1s examined, OsGL1D functions as a transcription activator. Our results also show that GUS activities were increased when *GL3*, but not *OsGL3B* was cotransfected with *GD-OsGL1D* (Fig. 5b), indicating that OsGL1D is able to interact with GL3, but not OsGL3B.

### Ectopic expression of *OsTTG1A* rescued *ttg1* phenotypes

After showing that OsGL1E, OsGL3B and OsTTG1A can form a MBW complex, we wanted to further examine if they may have similar functions as their Arabidopsis homologs. Considering that the *ttg1* mutant has a variety of obvious phenotypes relate to trichome and root hair cell fate determination and secondary metabolism including seed color, mucilage production and anthocyanin biosynthesis [12, 45, 46], and OsTTG1A showed high amino acid identity and similarity to TTG1, we decided to examine if OsTTG1A is a functional analogue of TTG1 by examine if ectopic expression of *OsTTG1A* could rescue the *ttg1* mutant phenotypes.

**Fig. 6:**
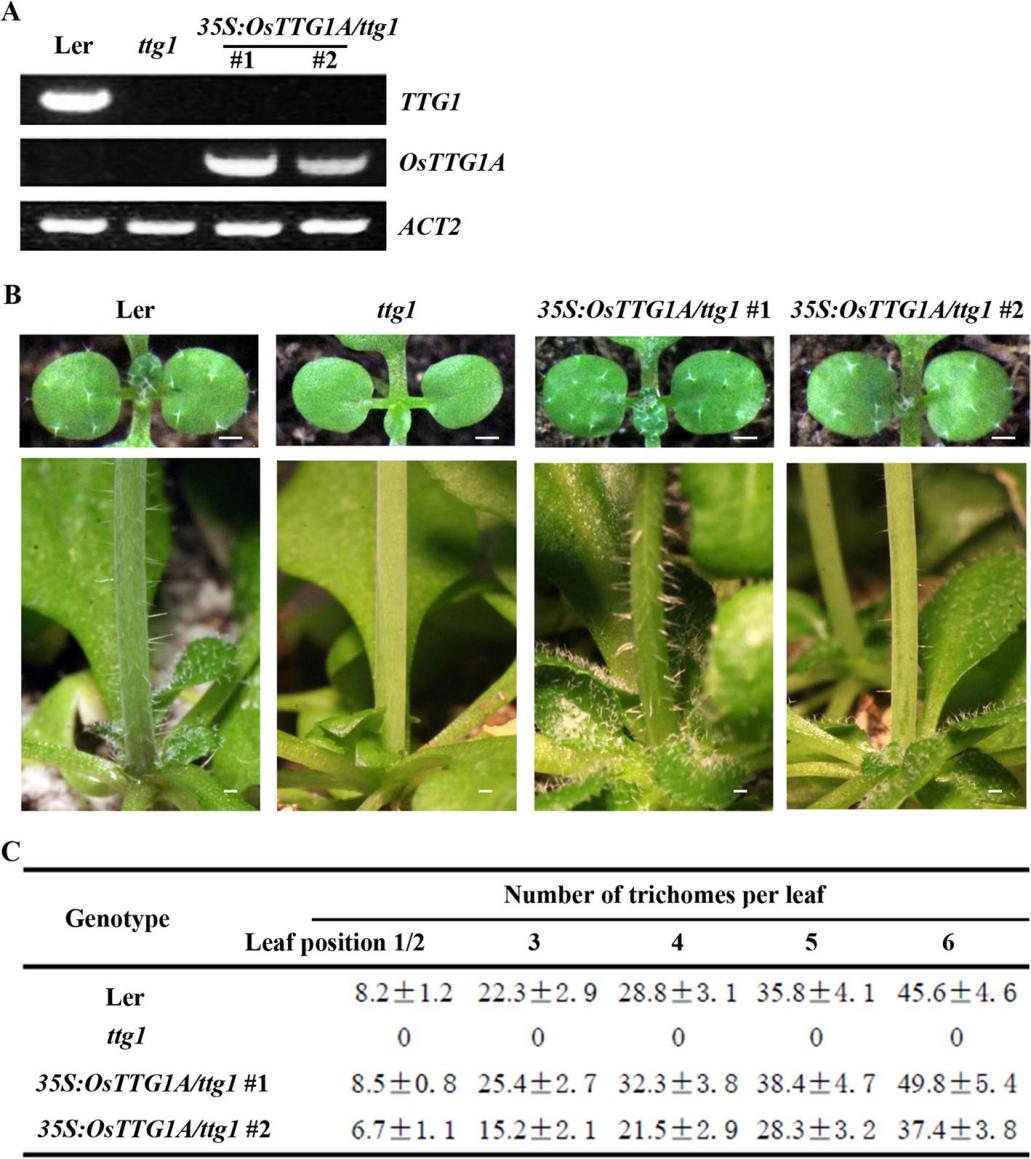
Ectopic expression of *OsTTG1A* restored trichome phenotype in the *ttg1* mutants. **a** Expression of *TTG1* and *OsTTG1A* in the Ler wild type, the *ttg1* mutant and the *35S:OsTTG1*/*ttg1* transgenic plants. RNA was isolated from 10-day-old seedlings, and RT-PCR was used to examine the expression of *TTG1* and *OsTTG1A*. The expression of *ACT2* was used as a control. **b** Trichome formation in leaves (up panel) and stems (lower panel) of the Ler wild type, the *ttg1* mutant and *35S:OsTTG1*/*ttg1* transgenic plants. Bar, 1 mm. **c** Number of leaf trichomes in the Ler wild-type, the *ttg1* mutant and *35S:OsTTG1*/*ttg1* transgenic plants. Data represent the mean ± SD of 12 plants

**Fig. 7:**
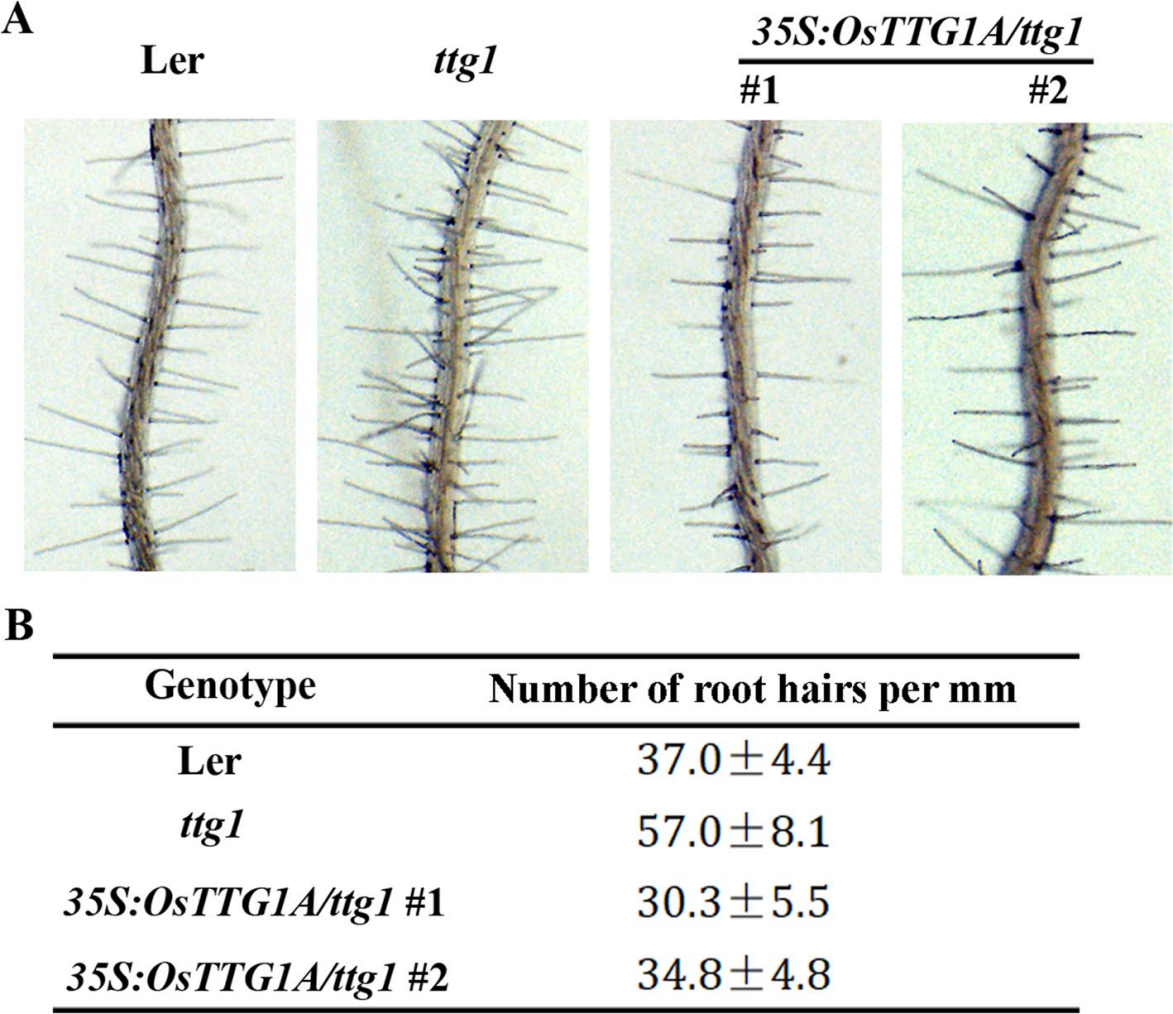
Ectopic expression of *OsTTG1A* restored root hair phenotype in the *ttg1* mutants. **a** Root hair formation in the Ler wild-type, the *ttg1* mutant and *35S:OsTTG1*/*ttg1* transgenic plants. **b** Number of root hairs in the Ler wild-type, the *ttg1* mutant and *35S:OsTTG1*/*ttg1* transgenic plants. Data represent the mean ± SD of at least 15 seedlings

Transgenic plants were generated in the *ttg1* mutant plants by expressing *OsTTG1A* under the control of the *35S* promoter (*35S:OsTTG1A/ttg1*). Two independent homozygous lines were used for phenotypic analysis. As shown in Fig. 6a, transcript of *TTG1* was only detectable in the Ler wild type plants, whereas transcript of *OsTTG1A* was only detectable in the *35S:OsTTG1A/ttg1* transgenic plants, and relative high transcript level of *OsTTG1A* was observed in seedlings of the *35S:OsTTG1A/ttg1* #1 line. We observed that plants of both *35S:OsTTG1A/ttg1* transgenic lines produced trichomes on rosette leaves and stems (Fig. 6b). Quantitative analysis showed that plants of the *35S:OsTTG1A/ttg1* #1 line produced more trichomes on rosette leaves (Fig. 6c), consistent with the relatively high transcript level in seedlings of this line. On the other hand, reduced root hairs formation was observed in both of the *35S:OsTTG1A/ttg1* transgenic lines when compared with the *ttg1* mutants (Fig. 7a), and quantitative analysis showed that root hair density in the *35S:OsTTG1A/ttg1* transgenic seedlings is similar to the Ler wild type (Fig. 7b).

**Fig. 8:**
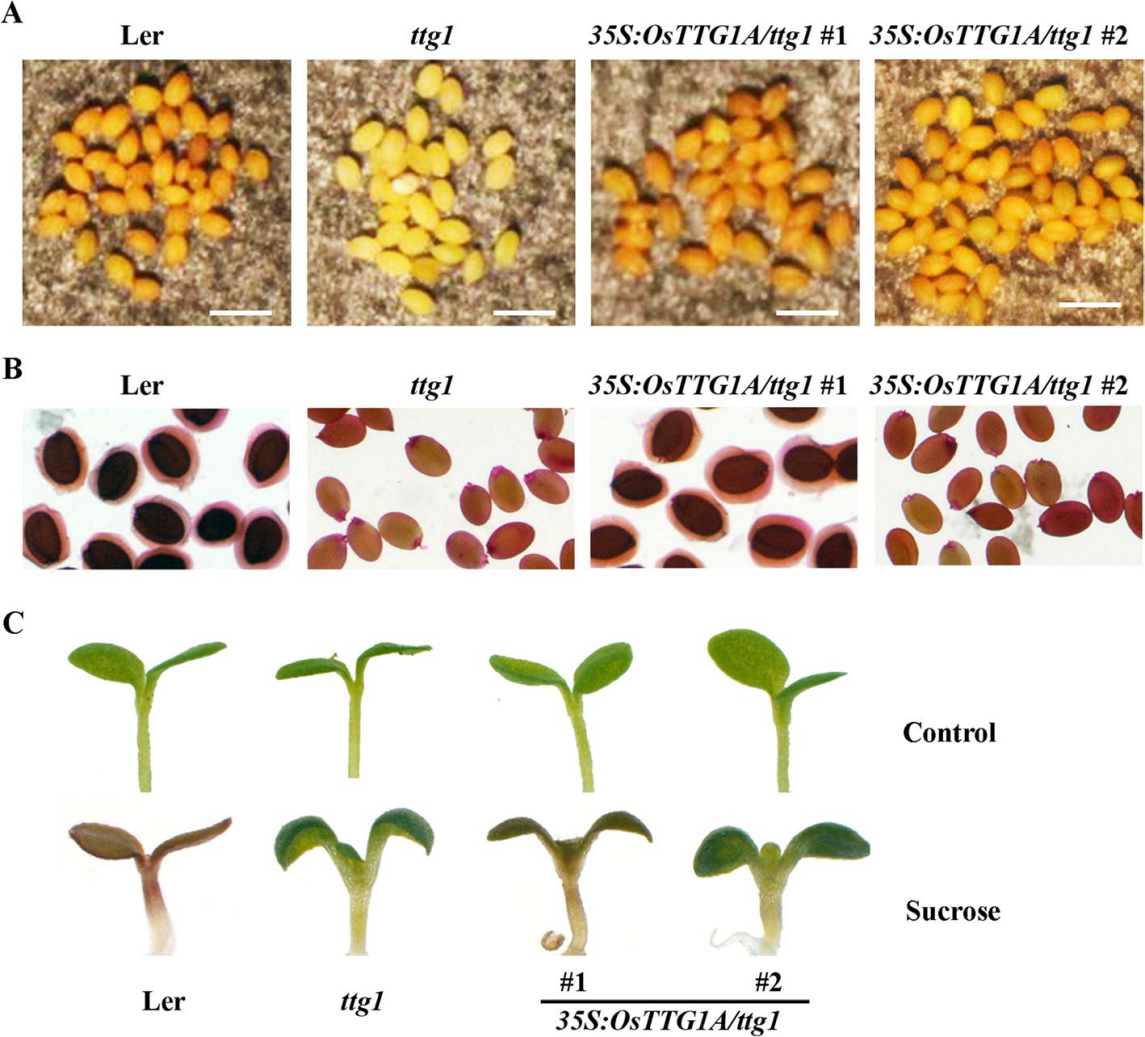
Ectopic expression of *OsTTG1A* restored secondary metabolism phenotype in the *ttg1* mutants. **a** Seed coat color in the Ler wild type, the *ttg1* mutant and *35S:OsTTG1*/*ttg1* transgenic plants. Bar, 1 mm. **b** Mucilage production in the Ler wild type, the *ttg1* mutant and *35S:OsTTG1*/*ttg1* transgenic plants. **c ** Anthocyanin biosynthesis in the Ler wild type, the *ttg1* mutant and *35S:OsTTG1*/*ttg1* transgenic plants

The seed color phenotype of the *ttg1* mutant was recovered in the *35S:OsTTG1A/ttg1* transgenic plants, but also to different degree in the two different lines (Fig. 8a). Whereas mucilage production in the *35S:OsTTG1A/ttg1* #1 line was nearly similar to the Ler wild type, but that in #2 line was largely similar to the *ttg1* mutants (Fig. 8b), anthocyanin biosynthesis was also largely recovered in the *35S:OsTTG1A/ttg1* #1 line, but not #1 line seedlings (Fig. 8c). These results indicate that OsTTG1A is likely the functional analogue of TTG1.

**Fig. 9:**
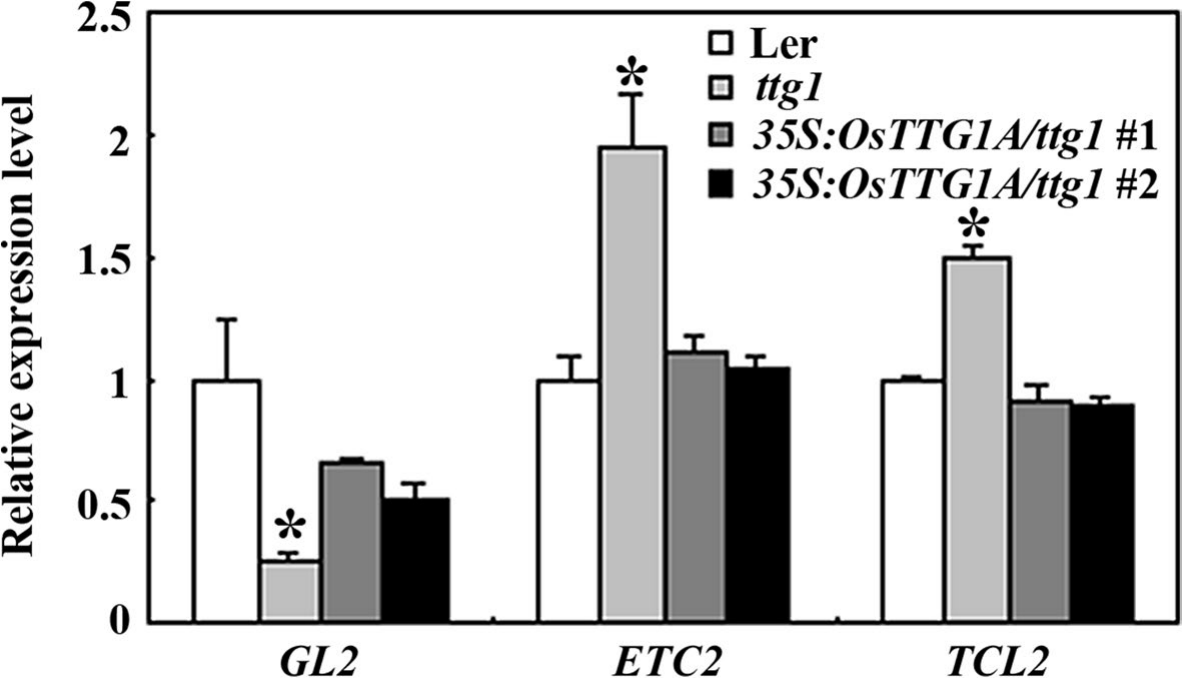
Expression of trichome formation regulating genes downstream of TTG1 in the *35S:OsTTG1A/ttg1* transgenic plants. RNA was isolated from 10-day-old seedlings of the Ler wild type, the *ttg1* mutants, and the *35S:OsTTG1A/ttg1* transgenic plant seedlings and qRT-PCR was used to examine gene expression. *ACT2* was used as an inner control, and the expression level of the corresponding genes in the Ler wild type seedings was set as 1. Data represent the mean ± SD of three biological replicates. *: Significantly different from that in the Ler wild type (Student’s *t*-test *P* < 0.05)

We then examined the expression of the TTG1 down stream trichome formation regulator genes in the Ler wild type, the *ttg1* mutant and *35S:OsTTG1A/ttg1* transgenic plant seedlings, including *GL2* and the R3 MYB genes. We found that the expression level of *GL2* was significantly reduced, whereas that of *ETC2* and *TCL2* increased in the *ttg1* mutants, whereas that in the *35S:OsTTG1A/ttg1* transgenic plants were largely similar to the Ler wild type seedlings (Fig. 9).

## Discussion

A MYB-bHLH-WD40 (MBW) complex formed by the R2R3 MYB transcriptional activator GL1, the bHLH transcription factor GL3, EGL3 or TT8, and the WD40-repeat protein TTG1 regulates trichome initiation in Arabidopsis [3, 4, 6, 7, 24]. By identifying and characterizing rice homologs of the trichome initiation-regulating MBW complex proteins, we found that similar MBW complex may present in rice and at least some components in the complex may have similar functions as the ones in Arabidopsis.

First, the rice homologs shared similar features as the Arabidopsis MBW complex, i.e., the [D/E]L × 2[R/K] × 3L × 6L × 3R amino acid signature required for the interaction of MYB transcription factors with bHLH transcription factors [41], and the S residue required for activation of *GL2* [42], are full conserved in all the five OsGL1s (Fig. S1); The first 97 amino acids region in GL1 that is required for its interaction with GL3 [9], is highly conserved in both OsGL1A and OsGL1B (Fig. S2); and the 25 amino acids region in TTG1 that is required for its interaction with GL3 [9], is also highly conserved in OsTTG1A and OsTTG1B (Fig. S3). Second, it has been shown that GL3 functions as a transcription activator, but GL1 and TTG1 do not [41, 44], similar, our results show that OsGL3B activated reporter gene expression in transfected protoplast, but OsGL1E and OsTTG1 failed to do so (Fig. 3). Third, OsGL3B interacted with OsGL1E and OsTTG1A, respectively (Figs. 3 and 4), indicating that they can form a MBW complex. In addition, OsGL1E and OsTTG1A can interact with GL3 (Figs. 3 and 4), and OsGL3B interacted with GL1 and TTG1 (Fig. 5), suggested that rice homologs are interchangeable with their Arabidopsis MBW complex proteins in forming MBW complexes. Last but not least, ectopic expression of *OsTTG1A* restored the phenotypes in the *ttg1* mutant (Figs. 6, 7 and 8), indicating that OsTTG1A is likely the functional analogue of TTG1 in controlling trichome formation in Arabidopsis. Considering that OsGL1E and OsGL3B are able to form MWB complex with OsTTG1A, it may be worthwhile to examine if they have similar functions with GL1 and GL3, respectively in regulating trichome formation in Arabidopsis. Since OsTTG1A and OsTTG1B show high amino acid sequence similarly, it will be of interest to examine if OsTTG1B also have similar functions. Considering that OsGL3A is closely related to OsGL3B, it will be also of interest to examine if OsGL3A may form MBW complex with OsGL1s and OsTTG1A.

The MBW complex formed by GL1, GL3/EGL3 and TTG1 regulates trichome initiation via activating *GL2* [3–5, 7], but the same MBW complex can also activate the R3 MYB trichome initiation inhibitor genes [17–22]. Even though our previously study showed that OsTCL1 can regulate trichome initiation in Arabidopsis, but it can not in rice [40], considering that trichome initiation in rice can be regulated by OsWOX3B, a homolog of GL2 [33–35], it is worthwhile to examine if MBW complex formed by OsGL1E, OsGL3B and OsTTG1A is able to regulate trichome initiation in rice via activating *OsWOX3B*.

As discussed above, some of our experiments support that rice homologs and the Arabidopsis MBW complex proteins may have conserved functions, but some others also suggest that they may also have non-conserved functions. First, OsGL1A and OsGL1B shared higher amino acid sequence identity and similarity with GL1 when compared with OsGL1D and OsGL1E (Fig. S1), however, they can not interact with GL3 or OsGL3B (Figs. 3  and 4). Considering that the [D/E]L × 2[R/K] × 3L × 6L × 3R amino acid signature and the S residue required are conserved in all the five OsGL1s (Fig. S1), it is very likely the some other amino acid residues may also be critical for the interaction of OsGL1s/GL1 with GL3/OsGL3B. According to the amino acid sequence alignment, in the R3 MYB domain of OsGL1A and OsGL1B, there are only a few amino acids are different from these in GL1 and OsGL1E, it will be interest to examine if any of them may be critical for the interaction of OsGL1s/GL1 with GL3/OsGL3B. Second, unlike GL1, OsGL1D functions as a transcription activator (Fig. 3). It is unlikely that OsGL1D contains an activation domain, whereas other OsGL1s do not, since previously we have shown that the C-terminal domain of GL1 confer transcriptional activation activities, but as a whole, GL1 does not function as a transcription activator [44]. Therefore, it is worthwhile to figure out why OsGL1D as a whole, is able to show its transcriptional activation activities, whereas GL1 and other OsGL1s can not. Third, OsGL1D interacted with GL3, but not OsGL3 (Figs. 3 and 4). Considering that OsGL1D is closely related to the OsGL1E (Fig. 1), it will also be interesting to examine why OsGL1D can interact with GL3 but not OsGL3B.

## Conclusions

Our results show that OsGL1E, OsGL3B and OsTTG1A are able to form a MBW complex, and they are interchangeable with the Arabidopsis MBW complex proteins in forming MBW complexes. Further more, OsTTG1A is also interchangeable with TTG1 in regulating trichome initiation in Arabidopsis. Our results also show that OsGL1D is a transcription activator, and it can interact with GL3, but not OsGL3B. These results suggest that rice homologs and the Arabidopsis MBW complex proteins have conserved and non-conserved functions.

## Methods

### Bioinformatics analysis

Identification of GL1 and GL3 homologs in rice (*Oryza sativa*), including OsGL1A, OsGL1B, OsGL1C, OsGL3A, OsGL3B, OsGL3C, OsTTG1A, and OsTTG1B has been described previously [40]. OsGL1D and OsGL1E were identified as potentially interaction proteins of OsGL3B on STRING (https://string-db.org/).

Full-length amino acid sequences were used for phylogenetic analysis and sequence alignment. Phylogenetic analysis was performed on Phylogeny (www.phylogeny.fr) by using “One Click” mode with default settings. The analysis was performed by selecting “One Click” from the tab “Phylogeny Analysis” on the website, and then inputting the amino acid sequences as promoted. The details setting of “One Click” mode can be viewed by clicking “Overview” after the “One Click” mode was selected. Sequence alignment was generated by using BioEdit. Percentage of amino acid identity and similarity were calculated by using MatGAT (v2.02) [47].

### Plant materials and growth conditions

The Japonica rice variety *Nipponbare* was used for gene cloning, the Columbia-0 (Col) Arabidopsis was used for protoplasts isolation, and the Landsberg erecta (Ler) Arabidopsis was used as a control for phenotype analysis. The *ttg1* mutant was in the Ler ecotypic background [12].

For trichome phenotypic analysis, protoplast isolation and plant transformation, seeds of indicated Arabidopsis materials were germinated and grown in soil pots as described previously [48]. For root hair phenotypic analysis, seeds were surface-sterilized and sown on solidified 1/2 MS (Murashige and Skoog) medium and grown vertically in a growth room as described previously [40]. More than 15 seedlings for each line were used for trichome and root hair phenotype analysis.

For RNA isolation from Arabidopsis to examine the expression of *TTG1* and *OsTTG1A*, seeds of the Ler wild type, the *ttg1* mutant, and the *35S:OsTTG1A/ttg1* transgenic plants were surface-sterilized and sown on solidified 1/2 MS medium and grown vertically in a growth room as described previously [48]. For each genotype, a mix of at least eight seedlings was used for RNA isolation.

For RNA isolation from rice to clone related genes, seeds of *Nipponbare* rice were generated and grown in water in a growth room for 10 days. The photoperiod in the growth room was 16 h light/8 h dark, the light density was ~ 120 μmol m^−2^ s^−1^, and the temperature was 22 °C for Arabidopsis, and 28 °C for rice [40]. A mix of five seedlings was used for RNA isolation.

The seeds used for phenotypic analysis and examine the gene expression level were planted and collected in the same condition and same time.

### RNA isolation, RT-PCR and qRT-PCR

Ten-day-old Arabidopsis and rice seedlings were used for RNA isolation. Total RNA was isolated by using an EasyPure^TM^ Plant RNA Kit (Transgene Biotech) and following the manufacturer’s instructions. cDNA was synthesized by using an EasyScript First-Strand DNA Synthesis Super Mix (TransGen Biotech) and following the manufacturer’s procedures, and used for RT-PCR and qRT-PCR amplification. For expression analysis of *TTG1* and *OsTTG1A*, the expression of *ACTIN2* (*ACT2*) was used as a control. For expression of *GL2* and R3 MYB genes, the expression of *ACT2* was used as an inner control.

The primers used for amplification of *OsGL1A* are 5’-CAACATATGATGGGGAGGTCGCCGTGC-3’and 5’-CAACTTAAGTCATTTCATGGGGAGGCTTCTG-3’, for *OsGL1B* are 5’-CAACATATGATGGGGAGGTCACCG-3’and 5’-CAACTTAAGTCATTTCATTTCCAAGCTTCTG-3’, for *OsTTG1A* are 5’-CAACATATGGAGCAGCCCAAGCCG-3’ and 5’-CAACTTAAGTCAGACCCTGAGAAGCTGGA-3’, for *GL1* are 5’-CAACATATGAGAATAAGGAGAAGAGATGA-3’ and 5’-CAACTTAAGCTAAAGGCAGTACTCAACATC-3’, for *TTG1* are 5’-CAACATATGATGGATAATTCAGCTCCAGATTCG-3’ and 5’-CAACTTAAGTCAAACTCTAAGGAGCTGCATTTTG-3’, and for *GL3* are 5’-CAACATATGGCTACCGGACAAAACAG-3’ and 5’-CAAGAGCTCTCAACAGATCCATGCAACCC. Other primers used for RT-PCR have been described previously [40, 48].

### Constructs

The nuclear indicator construct *NLS-RFP*, the reporter construct *Gal4:GUS* and the effector constructs *GD* (Gal4 DNA Binding Domain), *CAT*, *GL3*, *GD-GL3*, *GD-GL1*, *GD-TTG1* used for protoplasts transfection have been described previously [44, 49, 50].

To make the HA (Human influenza hemagglutinin)-tagged *OsGL3B* construct, and GFP (Green fluorescent protein) tagged and/or GD-tagged *OsGL1s*, *OsGL3B* and *OsTTG1A* constructs for protoplast transfection, the full-length ORF (open-reading frame) of the corresponding genes were amplified by RT-PCR using RNA isolated from rice seedlings, or synthesized (for *OsGL3B*, *OsGL1C, OsGL1D* and *OsGL1E*) by Sangon Biotech Co., Ltd, and cloned in-frame with an N-terminal HA, GFP or GD tag and under the control of the double *35S* promoter into *pUC19* vector [50, 51].

To make *35S:OsTTG1A* construct for plant transformation, the HA tagged *OsTTG1A* construct in *pUC19* was digested with NdeI and AflII and subcloned into the binary vector *pPZP211* vector [52].

To generate bait and prey constructs for yeast-two-hybrid assays, *OsGL3B* was cloned into *pGBKT7* vector (Oebiotech), and *GL1* and *TTG1* and their homolog genes in rice were cloned into *pGADT7* vector (Oebiotech).

### Plant transformation and transgenic plants selection

For phenotype rescue experiment, the *35S:OsTTG1A* construct was introduced into *Agrobacterium tumefaciens* GV3101, and used to transform the *ttg1* mutant plants by using the floral dip method [53]. The *ttg1* mutant plants used for transformation were ~ 5-week-old, and have several mature flowers on the main inflorescence.

To select transgenic lines, T1 seeds were sown on 1/2 MS medium containing 50 μg/ml Kanamycin and 100 μg/ml Carbenicillin. More than 20 transgenic plants were obtained, and three trichome bearing T1 plants were chosen to isolate transgenic plants with a single T-DNA insertion locus in T2, and homozygous lines in T3 by germinating on 1/2 MS medium containing 25 μg/ml Kanamycin. Seeds from two homozygous lines were used for phenotypic analysis.

### Yeast two-hybrid assays

Yeast two-hybrid assay was performed by using Yeast Transformation System 2 (Clontech) according to the manufacturer’s instructions.

### Plasmid DNA isolation, protoplast transfection and GUS activity assay

Plasmid DNA used for protoplast transfection was isolated using a GoldHi EndoFree Plasmid Maxi Kit (CWBIO) according to the manufacturer’s instructions.

Protoplasts were isolated from 50 ~ 60 rosette leaves of 3 ~ 4-week-old Col plants, and transfected with plasmids of the reporter and effector genes by using the procedure described previously [50].

To examine subcellular localization of rice homologs of GL1, GL3 and TTG1, the plasmids of GFP fused constructs and nuclear indicator construct *NLS-RFP* were cotransfected into protoplasts. The transfected protoplasts were incubated at room temperature and under darkness for 20 ~ 22 h. GFP and RFP florescence were examined and photographed under a florescence microscope.

To examine the transcriptional activity of OsGL3B, plasmids of the reporter gene *Gal4:GUS* and the effector gene *GD-OsGL3B* were cotransfected into protoplasts. Cotransfections of *GD* and *GD-GL3* were used as negative and positive controls, respectively. To examine the possible interaction of OsGL3B with GL1 and TTG1 in plant cells, plasmids of the reporter gene *Gal4:GUS*, the effectors gene *GD-GL1* or *GD-TTG1*, and *OsGL3B* or *CAT* were cotransfected into protoplasts. To examine the possible interaction of OsTTG1A or OsGL1s with OsGL3B or GL3, plasmids of the reporter gene *Gal4:GUS*, the effector genes *GD-OsGL1s* or *GD-OsTTG1A*, and *OsGL3B*, *GL3* or *CAT* were cotransfected into protoplasts. Cotransfections of *GD* were used as controls. The transfected protoplasts were incubated at room temperature and under darkness for 20 ~ 22 h. GUS activities were measured using a microplate reader (Synergy™ HT, BioTEK). Transfection of each combination contains three biological replicates, and the experiments were repeated at least twice with similar results.

### Mucilage production assays

Seeds were stained and mounted as described previously [54], and mucilage was viewed examined under a Motic K dissecting microscope. At least 30 seeds were used for the assays.

### Anthocyanin biosynthesis assays

Anthocyanin biosynthesis was assayed as described previously [55], except that 5% rather than 3% sucrose was used for the experiment.

### Microscopy

GFP fluorescence in transfected protoplast was observed and photographs were taken under an Olympus FV1000 confocal microscope. Leaf trichome, root hair, mucilage, anthocyanin and seeds color were examined under a Motic K microscope, and photographs were taken using an EOS 1100D digital camera connected to the microscope. Photographs of stem trichomes were taken using an EOS 1100D digital camera.

## Supplementary Information

**Additional file 1: Fig. S1** Amino acid sequence alignment of GL1 and OsGL1s. Full-length amino acid sequences of GL1 and OsGL1s were used for sequence alignment by using BioEdit. Identify amino acids are shaded in black, and similar ones in gray. Underlines indicate the R2R3 MYB domain. The conserved amino acid signature [D/E]Lx2[R/K]x3Lx6Lx3R that is required for interaction between MYB proteins and R/B-like BHLH transcription factors are indicated by arrowheads. The S has been shown to be required for the interaction of GL1 with GL3/EGL3 is indicated by star.

**Additional file 2: Fig. S2 **Amino acid sequence alignment of GL3, EGL3 and OsGL3s.Full-length amino acid sequences of GL3, EGL3 and OsGL3s were used for sequence alignment by using BioEdit. Identity amino acids are shaded in black, and similar ones in gray. Black underlines indicate the HLH domain. Red underlines indicate the 97 amino acid sequence required for the interaction of GL3 with GL1.

**Additional file 3: Fig. S3** Amino acid sequence alignment of TTG1 and OsTTG1s.Full-length amino acid sequences of TTG1 and OsTTG1s were used for sequence alignment by using BioEdit. Identity amino acids are shaded in black, and similar ones in gray. Underline indicates the 25 amino acid sequence required for the interaction of TTG1 with GL3.

**Additional file 4: Fig. S4 **Phylogenetic tree of GL1, MYB23, MYB82, OsGL1s and GL1 homologs from other eight plant species. The entire amino acid sequences of GL1, MYB23, MYB82, OsGL1s and GL1 homologs from the Brassicaceae family plants *Brassica rapa*, *Capsella grandiflora* and *Capsella rubella*, the Fabidae family plant *Glycine max*, the Malpighiales family plant *Populus trichocarpa*, and the Panicoideae family plants* Zea mays*,* Setaria italica* and *Panicum hallii* were used for phylogenetic analysis on Phylogeny (www.phylogeny.fr) by using “One Click” mode with default settings. The number above the branch indicates branch support values. Bar indicates branch length.

**Additional file 5: Fig. S5** Phylogenetic tree of GL3, EGL3, TT8, OsGL3s and GL3 homologs from other eight plant species. The entire amino acid sequences of GL3, EGL3, TT8, OsGL3s and GL3 homologs from the Brassicaceae family plants *Brassica rapa*, *Capsella grandiflora* and *Capsella rubella*, the Fabidae family plant *Glycine max*, the Malpighiales family plant *Populus trichocarpa*, and the Panicoideae family plants* Zea mays*,* Setaria italica* and *Panicum hallii* were used for phylogenetic analysis on Phylogeny (www.phylogeny.fr) by using “One Click” mode with default settings. The number above the branch indicates branch support values. Bar indicates branch length.

**Additional file 6: Fig. S6. **Phylogenetic tree of TTG1, OsTTG1s and TTG1 homologs from other eight plant species. The entire amino acid sequences of TTG1, OsTTG1s and TTG1 homologs from the Brassicaceae family plants *Brassica rapa*, *Capsella grandiflora* and *Capsella rubella*, the Fabidae family plant *Glycine max*, the Malpighiales family plant *Populus trichocarpa*, and the Panicoideae family plants* Zea mays*,* Setaria italica* and *Panicum hallii* were used for phylogenetic analysis on Phylogeny (www.phylogeny.fr) by using “One Click” mode with default settings. The number above the branch indicates branch support values. Bar indicates branch length.

## Data Availability

All data generated or analyzed during this study are included in this published article.

## Abbreviations

Col: Columbia-0
GD: Gal4 DNA Binding Domain
GFP: Green fluorescent protein
HA: Human influenza hemagglutinin
Ler: Landsberg erecta
MBW: MYB-bHLH-WD40
MS: Murashige and skoog
ORF: Open-reading frame
PEG: Polyethyleneglycol
SD-TL: SD-Trp-Leu
SD-TLH: SD-Trp-Leu-His
SD-TLHA: SD-Trp-Leu-His-Ade
WT: Wild type
