# Correction to: Conserved and non‑conserved functions of the rice homologs of the Arabidopsis trichome initiation‑regulating MBW complex proteins

**DOI:** 10.1186/s12870-021-03074-7

**Published:** 2021-06-17

**Authors:** Kaijie Zheng, Xutong Wang, Yating Wang, Shucai Wang

**Affiliations:** 1 Key Laboratory of Soybean Molecular Design Breeding, Northeast Institute of Geography and Agroecology, Chinese Academy of Sciences, Changchun, China; 2 Key Laboratory of Molecular Epigenetics of MOE, Institute of Genetics and Cytology, Northeast Normal University, Changchun, China; 3 Laboratory of Plant Molecular Genetics & Crop Gene Editing, School of Life Sciences, Linyi University, Linyi, China

**Correction to: BMC Plant Biol 21, 234 (2021)**

**https://doi.org/10.1186/s12870-021-03035-0**

Following publication of the original article [1], Authors found a minor error in the equal contributors’ statement. “Kaijie Zheng and Xutong Wang contributed equally to this work‘’ was missing.

The original article has been corrected.
