# Supplementary figures and images for: Conserved and non-conserved functions of the rice homologs of the Arabidopsis trichome initiation-regulating MBW complex proteins
Source: BMC Plant Biol. 2021 May 25;21:234. doi: 10.1186/s12870-021-03035-0 (PMC8145838; doi:10.1186/s12870-021-03035-0)

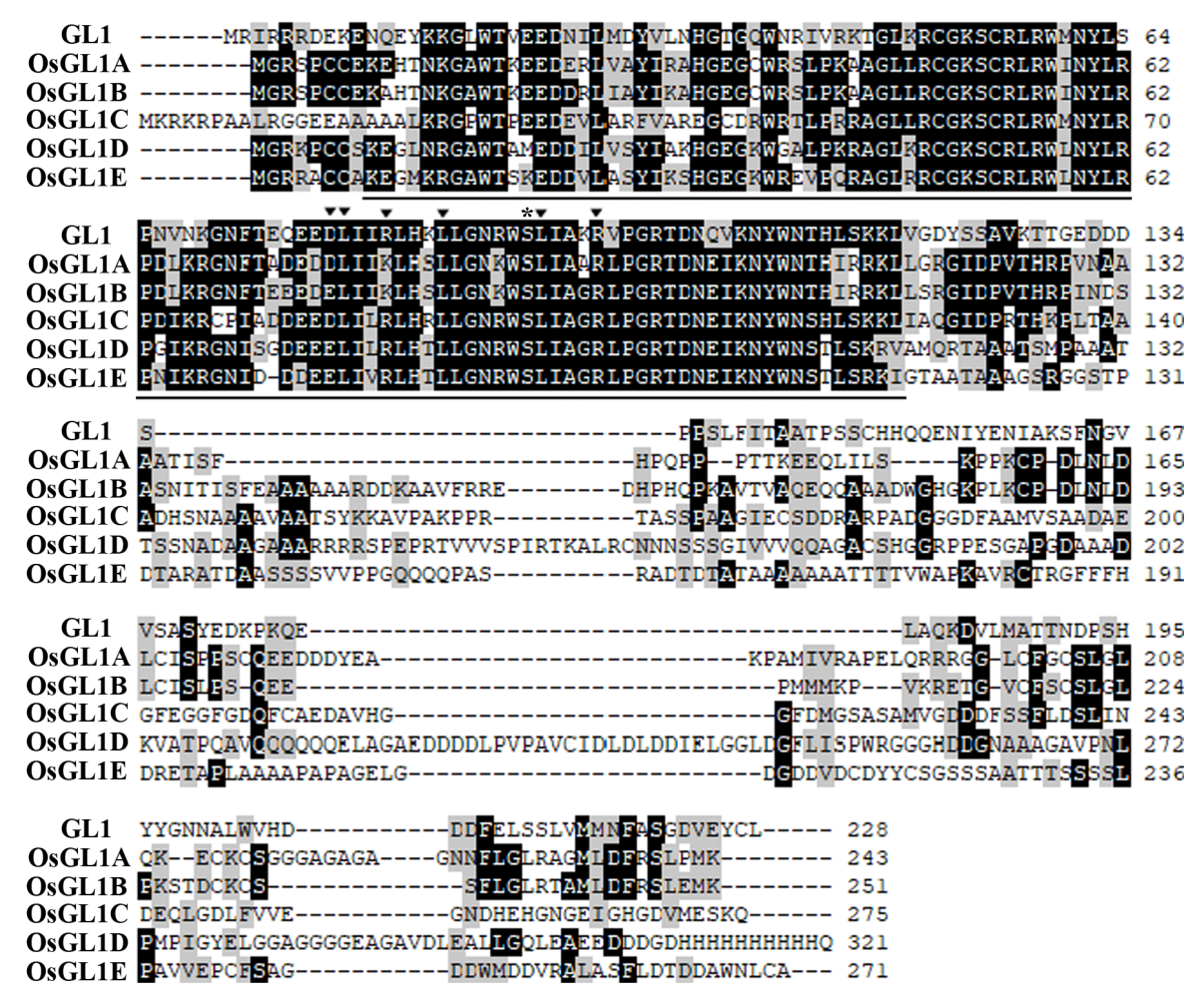


**Fig. S1**

Supplement: Supplementary file 1 — Additional file 1: Fig. S1 Amino acid sequence alignment of GL1 and OsGL1s. Full-length amino acid sequences of GL1 and OsGL1s were used for sequence alignment by using BioEdit. Identify amino acids are shaded in black, and similar ones in gray. Underlines indicate the R2R3 MYB domain. The conserved amino acid signature [D/E]Lx2[R/K]x3Lx6Lx3R that is required for interaction between MYB proteins and R/B-like BHLH transcription factors are indicated by arrowheads. The S has been shown to be required for the interaction of GL1 with GL3/EGL3 is indicated by star. [file 12870_2021_3035_MOESM1_ESM.docx]

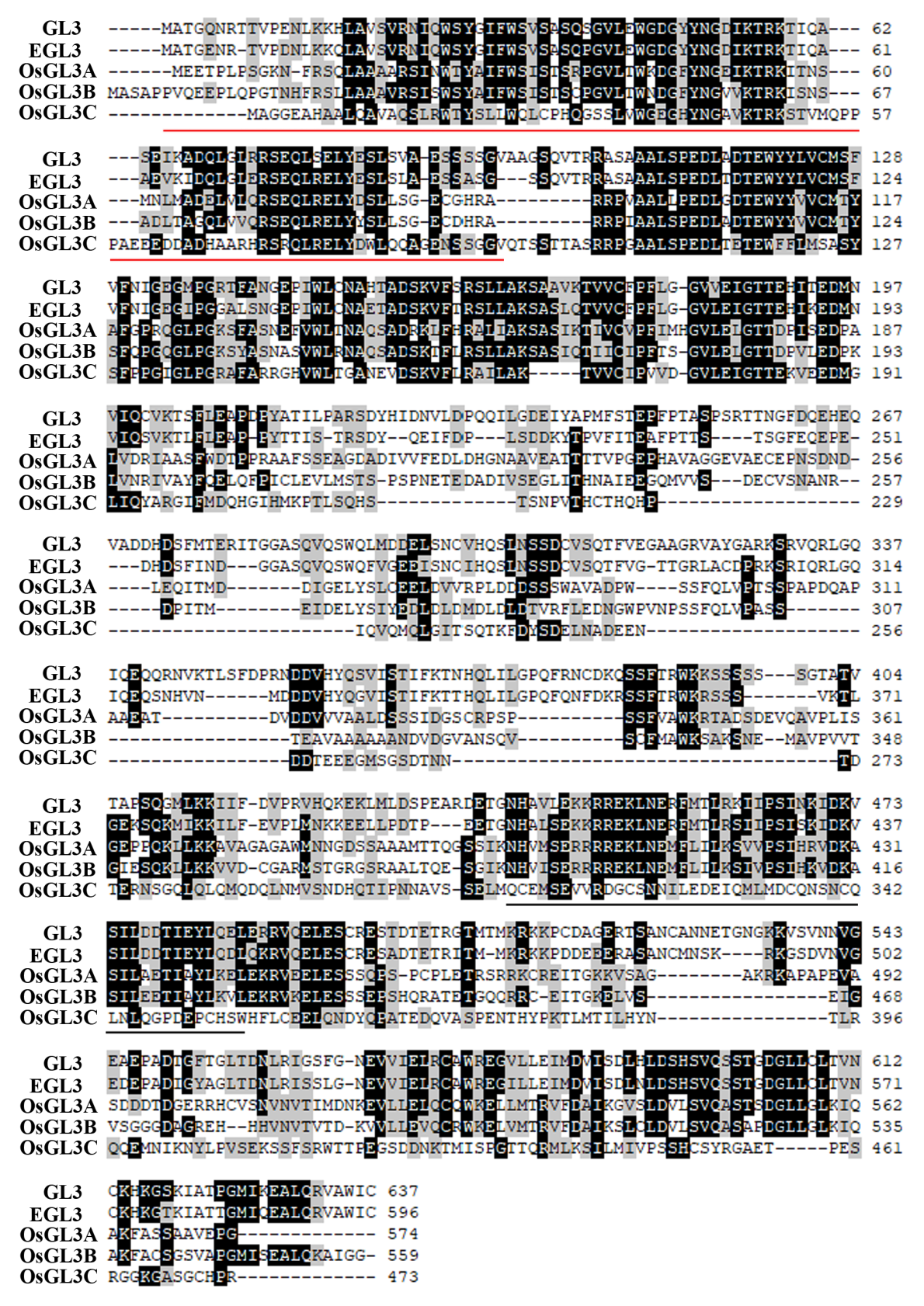


**Fig. S2**

Supplement: Supplementary file 2 — Additional file 2: Fig. S2 Amino acid sequence alignment of GL3, EGL3 and OsGL3s.Full-length amino acid sequences of GL3, EGL3 and OsGL3s were used for sequence alignment by using BioEdit. Identity amino acids are shaded in black, and similar ones in gray. Black underlines indicate the HLH domain. Red underlines indicate the 97 amino acid sequence required for the interaction of GL3 with GL1. [file 12870_2021_3035_MOESM2_ESM.docx]

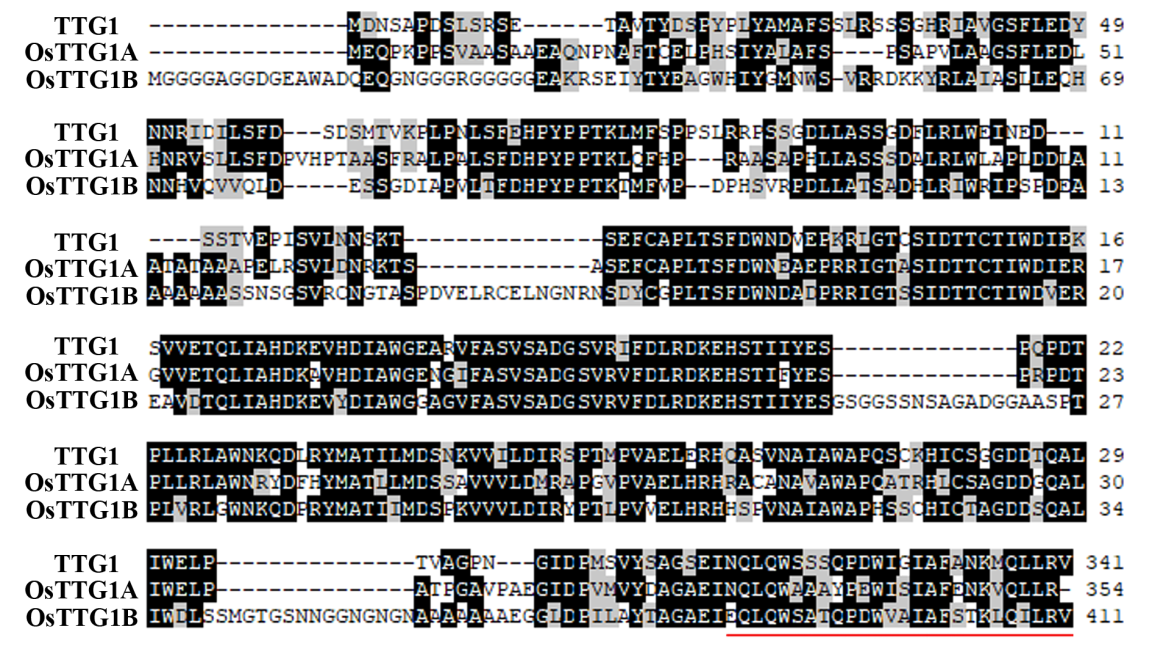


**Fig. S3**

Supplement: Supplementary file 3 — Additional file 3: Fig. S3 Amino acid sequence alignment of TTG1 and OsTTG1s.Full-length amino acid sequences of TTG1 and OsTTG1s were used for sequence alignment by using BioEdit. Identity amino acids are shaded in black, and similar ones in gray. Underline indicates the 25 amino acid sequence required for the interaction of TTG1 with GL3. [file 12870_2021_3035_MOESM3_ESM.docx]

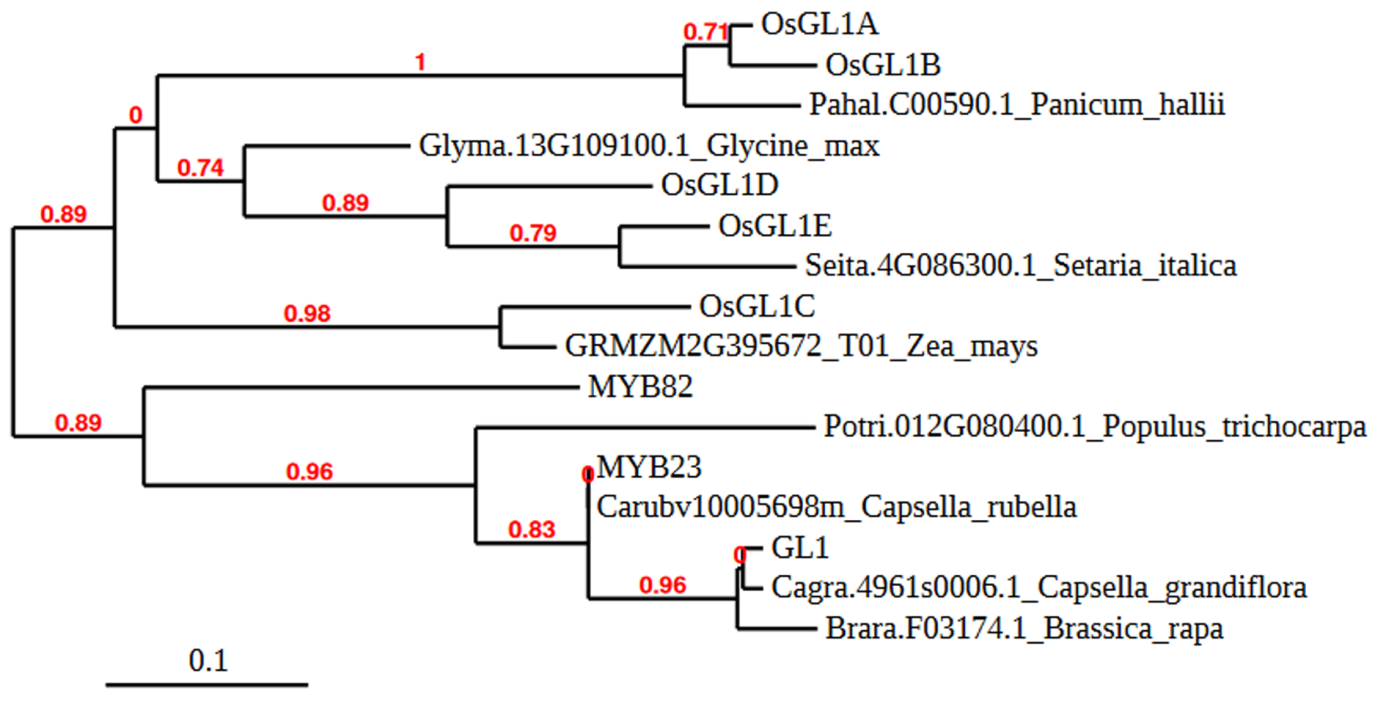


**Fig. S4**

Supplement: Supplementary file 4 — Additional file 4: Fig. S4 Phylogenetic tree of GL1, MYB23, MYB82, OsGL1s and GL1 homologs from other eight plant species. The entire amino acid sequences of GL1, MYB23, MYB82, OsGL1s and GL1 homologs from the Brassicaceae family plants Brassica rapa, Capsella grandiflora and Capsella rubella, the Fabidae family plant Glycine max, the Malpighiales family plant Populus trichocarpa, and the Panicoideae family plants Zea mays, Setaria italica and Panicum hallii were used for phylogenetic analysis on Phylogeny (www.phylogeny.fr) by using “One Click” mode with default settings. The number above the branch indicates branch support values. Bar indicates branch length. [file 12870_2021_3035_MOESM4_ESM.docx]

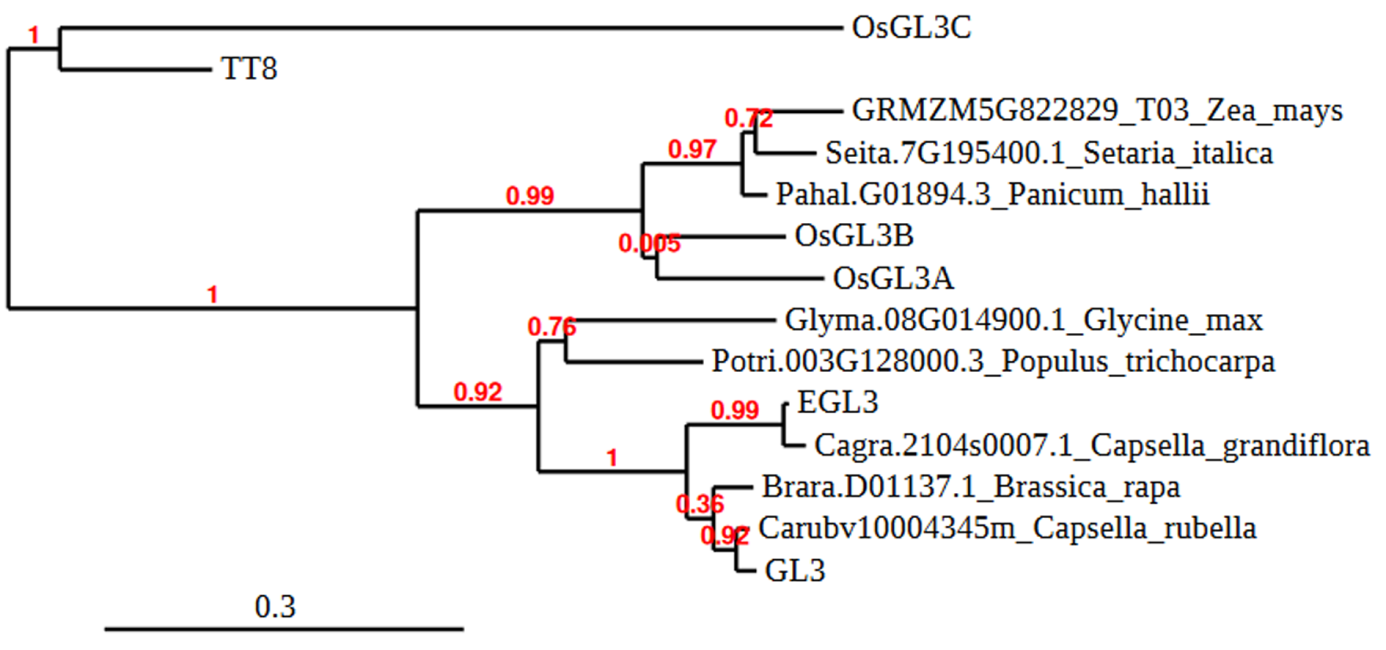


**Fig. S5**

Supplement: Supplementary file 5 — Additional file 5: Fig. S5 Phylogenetic tree of GL3, EGL3, TT8, OsGL3s and GL3 homologs from other eight plant species. The entire amino acid sequences of GL3, EGL3, TT8, OsGL3s and GL3 homologs from the Brassicaceae family plants Brassica rapa, Capsella grandiflora and Capsella rubella, the Fabidae family plant Glycine max, the Malpighiales family plant Populus trichocarpa, and the Panicoideae family plants Zea mays, Setaria italica and Panicum hallii were used for phylogenetic analysis on Phylogeny (www.phylogeny.fr) by using “One Click” mode with default settings. The number above the branch indicates branch support values. Bar indicates branch length. [file 12870_2021_3035_MOESM5_ESM.docx]

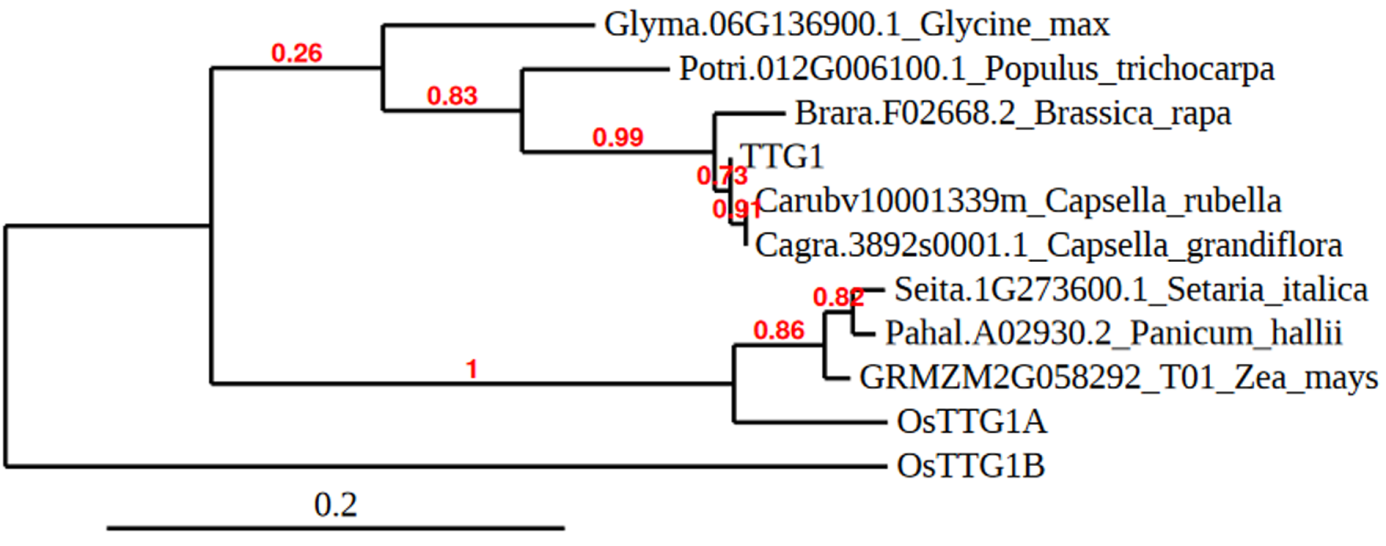


**Fig. S6**

Supplement: Supplementary file 6 — Additional file 6: Fig. S6. Phylogenetic tree of TTG1, OsTTG1s and TTG1 homologs from other eight plant species. The entire amino acid sequences of TTG1, OsTTG1s and TTG1 homologs from the Brassicaceae family plants Brassica rapa, Capsella grandiflora and Capsella rubella, the Fabidae family plant Glycine max, the Malpighiales family plant Populus trichocarpa, and the Panicoideae family plants Zea mays, Setaria italica and Panicum hallii were used for phylogenetic analysis on Phylogeny (www.phylogeny.fr) by using “One Click” mode with default settings. The number above the branch indicates branch support values. Bar indicates branch length. [file 12870_2021_3035_MOESM6_ESM.docx]
